# Inherited Metabolic Diseases and Cardiac Pathology in Adults: Diagnosis and Prevalence in a CardioMetabo Study

**DOI:** 10.3390/jcm9030694

**Published:** 2020-03-04

**Authors:** Marina Brailova, Guillaume Clerfond, Romain Trésorier, Régine Minet-Quinard, Julie Durif, Grégoire Massoullié, Bruno Pereira, Vincent Sapin, Romain Eschalier, Damien Bouvier

**Affiliations:** 1 Biochemistry and Molecular Genetic Department, CHU Clermont-Ferrand, 63000 Clermont-Ferrand, France; mplatonov@chu-clermontferrand.fr (M.B.); j_durif@chu-clermontferrand.fr (J.D.); 2 Cardiology Department, CHU Clermont-Ferrand, Faculty of Medicine, Université Clermont Auvergne, CNRS, SIGMA Clermont, Institut Pascal, 63000 Clermont-Ferrand, France; gclerfond@chu-clermontferrand.fr (G.C.); rtresorier@chu-clermontferrand.fr (R.T.); gmassoullie@chu-clermontferrand.fr (G.M.); reschalier@chu-clermontferrand.fr (R.E.); 3 INI-CRCT F-CRIN, 54500 Nancy, France; 4 Biochemistry and Molecular Genetic Department, CHU Clermont-Ferrand, Faculty of Medicine, Université Clermont-Auvergne, CNRS 6293, INSERM 1103, GReD, 63000 Clermont-Ferrand, France; rquinard@chu-clermontferrand.fr (R.M.-Q.); vsapin@chu-clermontferrand.fr (V.S.); 5 Biostatistics Unit (DRCI), CHU de Clermont-Ferrand, 63000 Clermont-Ferrand, France; bpereira@chu-clermontferrand.fr

**Keywords:** inherited metabolic disease, hypertrophic cardiomyopathy, arrhythmias, conduction disorders, prevalence, Fabry disease, SCAD, cardiac implantable electronic device

## Abstract

Many inherited metabolic diseases (IMD) have cardiac manifestations. The aim of this study was to estimate the prevalence of IMD in adult patients with hypertrophic cardiomyopathy (HCM) and cardiac rhythm abnormalities that require cardiac implantable electronic devices (CIEDs). The study included a review of the medical files of patients aged 18 to 65 years who were followed in our cardiology department during the period 2010–2017. Metabolic explorations for Fabry disease (FD), mitochondrial cytopathies, and fatty-acid metabolism disorders were carried out in patients with unexplained etiology. The prevalence of IMD in patients with HCM was 5.6% (confidence interval (CI): 2.6–11.6). Six cases of IMD were identified: 1 mitochondrial encephalopathy with lactic acidosis and stroke-like episodes (MELAS) syndrome, 1 Hurler syndrome, 2 Friedreich’s ataxia, 1 FD, and 1 short-chain acyl-CoA dehydrogenase deficiency. Three cases of IMD were identified in patients requiring CIEDs: 1 patient with Leber hereditary optic neuropathy, 1 FD, and 1 short chain acyl-CoA dehydrogenase (SCAD) deficiency. IMD prevalence in patients with CIEDs was 3.1% (CI: 1.1–8.8). IMD evaluation should be performed in unexplained HCM and cardiac rhythm abnormalities adult patients, since the prevalence of IMD is relatively important and they could benefit from specific treatment and family diagnosis.

## 1. Introduction

More than 40 inherited metabolic diseases (IMD) could have cardiac involvement, including fatty-acid oxidation defects, glycogen storage disorders, lysosomal storage disorders, peroxisomal disease, mitochondrial cytopathy, organic acidemias, aminoacidopathies, and congenital disorders of glycosylation [1]. Cardiac manifestations may reveal the disease or may appear during evolution, impacting the overall prognosis [2]. Cardiac manifestations in children are generally severe and sometimes fatal depending on the extent of metabolic deficit. Cardiac disease in adults could remain asymptomatic for a long time, may occur after a triggering factor (hypercatabolic situation, prolonged fasting, etc.), and may remain isolated. Early diagnosis is essential since some diseases respond to dietary modification and specific treatment [1].

The most common cardiac presentations are hypertrophic cardiomyopathy (HCM) and cardiac rhythm abnormalities. HCM is defined by the presence of increased left and/or right ventricular wall thickness that is not explained by abnormal loading conditions [3]. Cardiac hypertrophy could be primary or secondary to increased systemic vascular resistance [4]. The recommendations of the European Society of Cardiology (ESC) regarding HCM changed in 2014 [5]. The ESC “task force” is no longer limited to familial/sarcomeric HCM (40–60% of HCM), and the definition has extended to all potential HCM: 25–30% unknown cause and 5–10% genetic or nongenetic cause grouping metabolic diseases, neuromuscular diseases, and infiltrative cardiac diseases [3]. The etiological diagnosis of IMD and the management of patients have been the subject of new guidelines [5]. The overall prevalence of IMD among adults presenting a non-explicated HCM is not well established.

Ventricular arrhythmias, sudden cardiac death, and conduction disorders have been described in adult patients with fatty-acid oxidation defects, lysosomal disorders, and mitochondrial diseases in several case reports [1,6,7,8,9,10,11]. Sudden cardiac death may also be caused by progressive conduction disease in lysosomal storage disease and respiratory chain defects [1,2,9,10]. There are no studies on IMD prevalence in adult patients with severe arrhythmias and conduction disorders requiring cardiac implantable electronic devices (CIEDs; i.e., pacemaker (PM) and implantable cardioverter defibrillator (ICD)). In this context, the present CardioMetabo study evaluates the prevalence of IMD in a large cohort of patients diagnosed with HCM and/or for a CIED.

## 2. Experimental Section

### 2.1. Study Design

**Figure 1:**
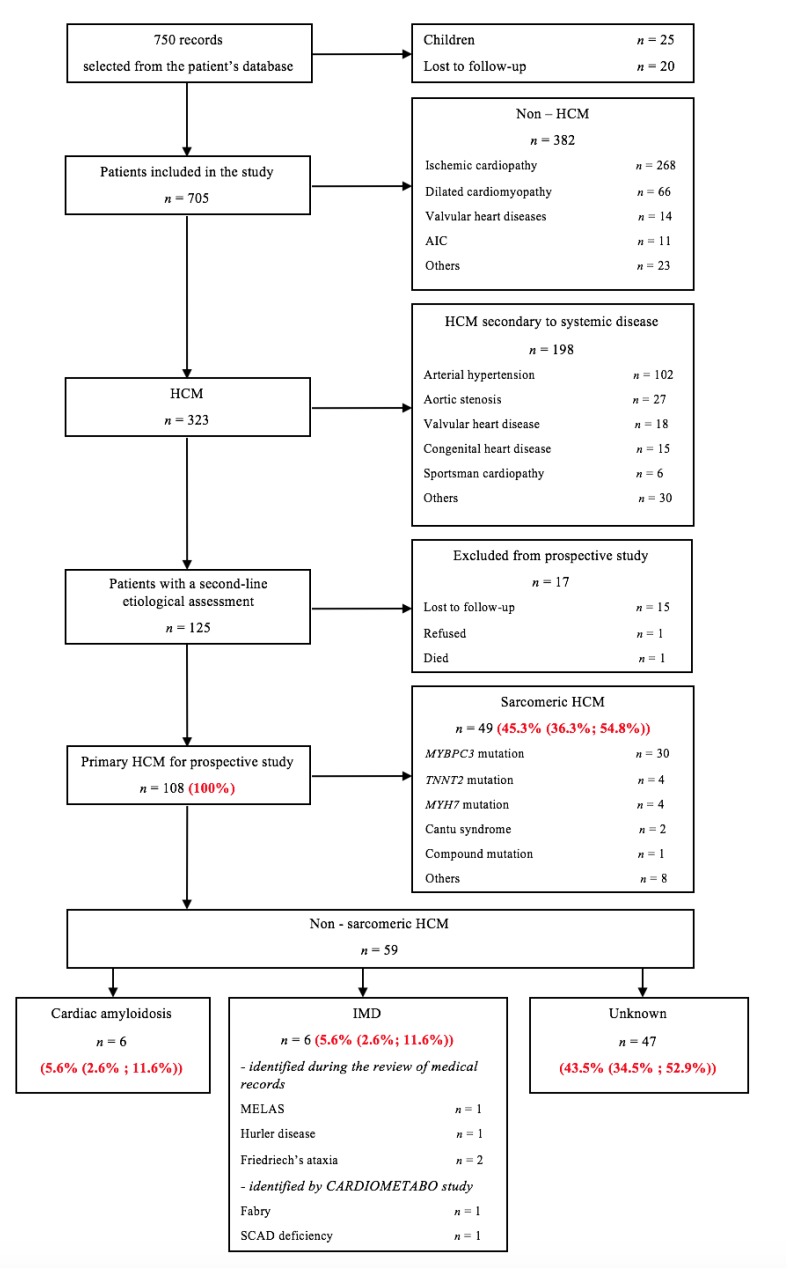
Study flow chart of patients with hypertrophic cardiomyopathy (HCM). AIC = arrhythmia induced cardiomyopathy; HCM = hypertrophic cardiomyopathy; IMD = inherited metabolic disease; MELAS = mitochondrial encephalopathy with lactic acidosis and stroke-like episodes; SCAD = short chain acyl-CoA dehydrogenase.

**Figure 2:**
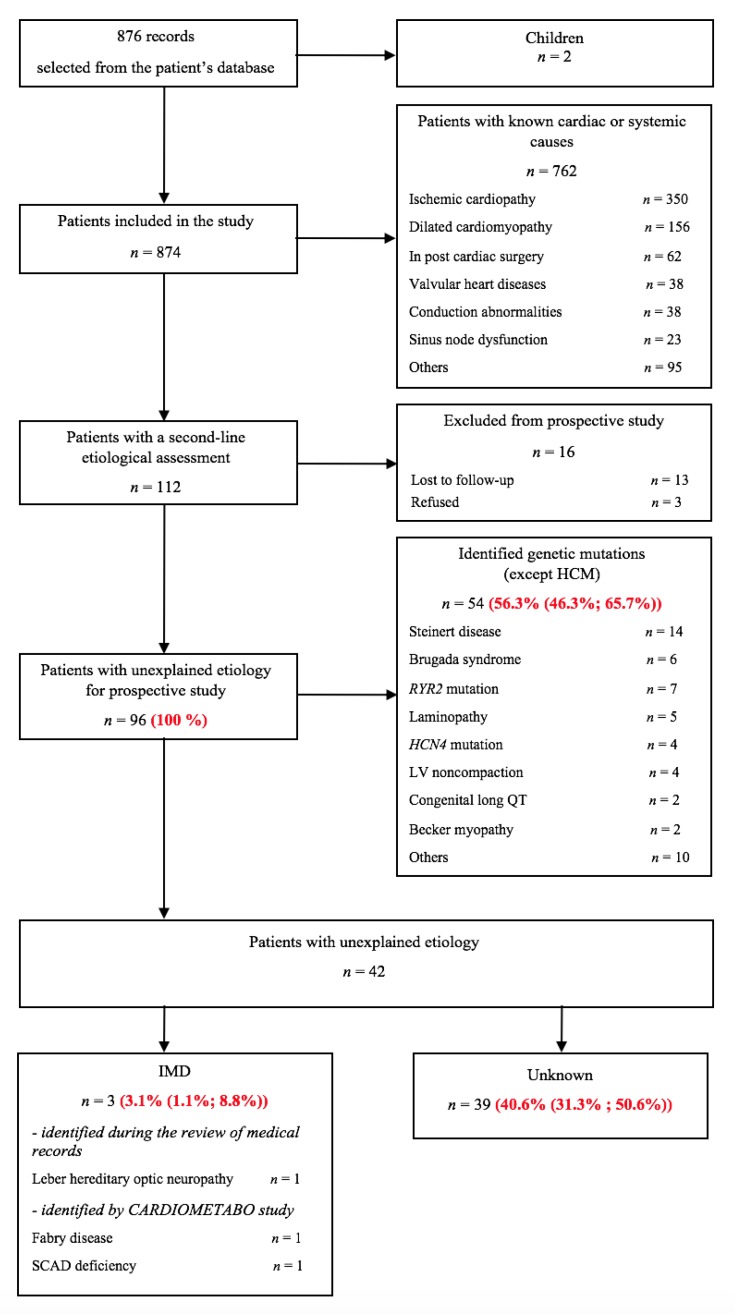
Study flow chart of patients with cardiac implantable electronic devices. HCM = hypertrophic cardiomyopathy; IMD = inherited metabolic disease; LV = left ventricle; QT = QT interval on the electrocardiogram; SCAD = short-chain acyl-CoA dehydrogenase.

CardioMetabo is a monocentric cohort study with retrospective and prospective phases. The flowcharts of patients are summarized in Figure 1 and Figure 2.

### 2.2. Retrospective Data Collection

The inclusions were realized from a database selection carried out by the Medical Information Department of the University Hospital of Clermont Ferrand over the period 2010–2017 according to the International Classification of Diseases, 10th Revision, Clinical Modification (ICD-10-CM) codes for HCM (I421, I422, or I517) [12] and Common Classification of Medical Acts codes for CIEDs (DELF005, DELF007, DELF015, DELF001, DELF016, DELF014, DELF020, or DELF013) [13] in patients aged 18–65 years. Two patient lists were obtained from the hospital database: the list of patients with HCM (+/− ICD or PM); and the list of patients with severe cardiac rhythm abnormalities requiring CIEDs (+/− HCM). The data collected were demographic characteristics (age, gender), cardiac-related medical history, electrocardiography, echocardiography, medications, results of biological and genetic analysis associated with heart disease, and results of screening and diagnosis of IMD.

### 2.3. Prospective CardioMetabo Study

The data analysis made it possible to define whether an etiology of HCM and cardiac rhythm abnormalities was initially identified. The patient files for which etiology could not be defined were selected to complete the metabolic investigation, and eventually the genetic study. According to the new ESC recommendations [3], the screening of Fabry disease, fatty-acid metabolism disorders, and mitochondrial cytopathy were part of the metabolic assessment to explain patients’ heart disease. 

### 2.4. Laboratory Tests

The diagnosis of Fabry disease in men was determined by measuring alpha-galactosidase A (αGAL) activity from dried blood spots on filter paper using tandem mass spectrometry (NeoLSD ^TM^ MSMS kit, Perkin-Elmer^®^, Turku, Finland). For the diagnosis of Fabry disease in women, in addition to testing of αGAL activity, we used the plasma globotriaosylsphingosine (lysoGb3) assessed by liquid chromatography/tandem mass spectrometry (LC-MS/MS) performed using LC20AD (Prominence Liquid Chromatograph, Shimadzu, Kyoto, Japan) coupled to an API 4500 QTrap (Applied Biosystems, Concord, ON, Canada). Patients with low αGAL activity or high lysoGb3 levels were submitted to a genetic study of the GLA gene by high-throughput sequencing on a NextSeq500^®^ sequencing platform (Illumina, San Diego, CA, USA).

The profile of acylcarnitines and the determination of total and free carnitine used for exploration of fatty-acid metabolism disorders were assayed by a flow injection analysis- tandem mass spectrometry with derivatization (FIA MS/MS) on API 3200TM (Applied Biosystems, Concord, ON, Canada) using an NSK-B Cambridge isotope. An investigation of cytoplasmic and mitochondrial redox potentials was carried out using enzymatic and spectrophotometric techniques. Routine investigations performed during the CardioMetabo study comprised creatinine, creatine kinase, troponin, NT-pro-BNP, and blood glucose levels, which were achieved using Dimension Vista^®^ 1500 (Siemens, Eschborn, Germany). In the event of missing results in the genetic assessment of HCM, a search for mutations was carried out. The genetic study of HCM genes panel *(MYBPC3, MYH7, MYL2, TNNI3, TNNT2)* was performed by high-throughput sequencing on a NextSeq500^®^ sequencing platform (Illumina, San Diego, CA, USA).

### 2.5. Ethics

The study was declared to the National Commission of Informatics and Freedoms and was submitted to the ethics review of the Committee for the Protection of Persons South East VI (approval reference no. 2018/CE02). An informed written consent was obtained and stored in the case of the genetic study.

### 2.6. Statistical Analysis

All the statistical analyses were performed using Stata software (version 13, StataCorp, College Station, TX, USA). The tests were two-sided, with a type-I error set at 5%. Categorical data were presented with numbers and percentages. The 95% CI of prevalence was estimated using the Wilson method, which appears more consistent and relevant for small sample sizes [14]. Continuous data were expressed as mean ± standard deviation (SD) or median (interquartile range (IQR)), according to the statistical distribution. The assumption of normality was assessed using the Shapiro–Wilk test.

Univariate analyses were then carried out in order to compare continuous variables between independent groups (HCM with IMD, sarcomeric HCM, unexplained HCM) using analysis of variance (ANOVA) or the Kruskal–Wallis test when the hypotheses of ANOVA were not met. In a second step, multivariable analyses were executed to take into account adjustment for age of patient, a possible confounder. More precisely, multiple linear regressions were realized applying a Sidak’s type-I error correction for multiple comparisons. The normality of residuals was studied as aforementioned. When appropriate, a logarithmic transformation was applied to assess the normality of the dependent variable, and guarantee that multivariable models were used appropriately. We used a Mann–Whitney U test to compare the median age of patients with primary HCM and patients with secondary left ventricular hypertrophy. We also used a Mann–Whitney test to compare the median age of patients with an identified etiology of cardiac rhythm abnormalities and patients with no identified etiology. 

## 3. Results

### 3.1. Hypertrophic Cardiomyopathy

Figure 1 presents the flowcharts of patients comprising the diagnostic work-up for HCM. A total of 750 medical records were reviewed from 2010 to 2017. Overall, 323 patients with HCM were eligible for the retrospective study (excluded: 25 children, 20 lost to follow-up, and 382 non-HCM). Secondary left ventricular hypertrophy was found for 198 patients with mostly arterial hypertension or aortic stenosis. After exclusion of 17 patients (lost to follow-up, refusal, death), 108 patients with primary HCM were eligible for a prospective study. For the subgroup of primary HCM, the median age of diagnosis of HCM was 39.5 years (IQR 22–51). This median age is significantly lower (*p* < 0.001) than the median age for patients with secondary left ventricular hypertrophy (53.5 years (IQR 46.7–59)).

Six patients had a diagnosis of amyloidosis, 5 with AL amyloidosis and 1 with transthyretin amyloidosis. The diagnoses respond to current recommendations [15]. For AL amyloidosis, 4 patients combine histological evidence of extracardiac amyloidosis with typical cardiac imaging. For the last patient, amyloidosis was showed on autopsy. For the patient with ATTR amyloidosis, it combined a positive Technetium scan, an absence of hematology abnormality and favorable cardiac imaging.

Sarcomeric mutation was found in 45.3% of patients (CI: 36.3–54.8). Four patients with IMD were reported during the review of medical records: 1 patient with MELAS (m.3243A > G), 1 patient with Hurler disease (p.P533R/p.E178K in *IDUA* gene), and 2 patients with Friedreich’s ataxia (expansion of the number of GAA repeats in exon 1 of *FXN* gene). The CardioMetabo assessment permitted us to newly diagnose 2 IMD: 1 patient with Fabry disease and 1 patient with short-chain acyl-CoA dehydrogenase (SCAD) deficiency. The clinical characteristics of patients with IMD are shown in Table 1. In patient with Fabry disease, a new missense mutation was identified (p.M96I in *GLA* gene). Genetic testing for SCAD deficiency was not performed due to the patient’s refusal.

The prevalence of IMD in patients with HCM was 5.6% (CI: 2.6–11.6). Cardiac amyloidosis was the cause of cardiac disease in 5.6% of patients (CI: 2.6–11.6). HCM etiology remained unknown in 43.5% of patients (CI: 34.5–52.9).

### 3.2. Cardiac Rhythm Abnormalities Requiring CIEDs

Figure 2 presents the flowcharts of patients comprising the diagnostic work-up for patients with CIEDs. A total of 876 medical records of patients with CIEDs were selected from the hospital database. Two children were excluded, so the population eligible for the retrospective study was made up of 874 individuals. An etiology of cardiac rhythm abnormalities was identified in 762 patients: Ischemic heart disease in the majority of cases, but also dilated cardiomyopathy, complication of surgery, conduction abnormalities, or sinus node dysfunction. In this group, the median age of cardiac device implantation was 58 years (IQR 52–62). After excluding 16 patients (lost to follow-up, refusal), 96 patients were eligible for the prospective study. The median age of cardiac electronic device implantation in this group was 44.5 years (IQR 30–54.7). Patients included in the prospective study were significantly younger (*p* < 0.0001). Of the 96 patients we studied, a mutation associated with an inherited cardiac arrhythmia was identified in 54 cases (56.3 % (CI: 46.3–65.7)). One patient with IMD had been reported during the review of the medical records (Leber hereditary optic neuropathy associated with the G > A mtDNA point mutation). The CardioMetabo assessment permitted us to newly diagnose 2 IMD: 1 patient with Fabry disease and 1 patient with SCAD deficiency (Table 1). The prevalence of IMD in patients with CIEDs was 3.1% (CI: 1.1–8.8). Severe cardiac rhythm abnormalities remained unexplained in 40.6% cases (CI: 31.3–50.6).

### 3.3. Biological Data Study

We compared the results of biochemical analyses in blood of patients with IMD with the patients presenting sarcomeric mutation detected in the study and the patients with an unexplained etiology (Table 2). The results presented in Table 2 relate to all patients who underwent the CardioMetabo assessment. The patients with IMD were younger than other individuals (*p* = 0.026). NT-pro-BNP and troponin plasma levels in patients with IMD were higher than in patients with an unexplained etiology (*p* = 0.043). The proportion of positive troponin value among patients with IMD was 71.43%, 12.5% among those with unexplained etiology (*p* = 0.005), and 14.29% among patients with sarcomeric mutations. These three groups did not differ significantly in terms of other biochemical parameters (free and total carnitine, lactic acid, pyruvic acid, creatine kinase, glucose).

## 4. Discussion

Inherited metabolic diseases are often an unknown cause of cardiac diseases in adults. Their prevalence in the pediatric population is well studied [1]. However, there has been no work to study the prevalence of hereditary metabolic diseases in adults with two types of cardiac disease: HCM and severe cardiac rhythm abnormalities requiring CIEDs. For the first time, the present study (CardioMetabo) highlighted a relative high proportion of IMD in such a population (5.6% and 3.1% in unexplained HCM and CIEDs, respectively). For the 6 patients with IMD and HCM, all HCM were diagnosed in adulthood. In a third of the cases, the IMD was diagnosed in childhood. In two thirds of the cases, the cardiac pathology preceded the diagnosis of IMD, carried out in adulthood. Our study established that patients selected for IMD research by the CardioMetabo assessment were significantly younger than the overall study population. This result is in accord with the ESC guidelines, which recommend a diagnostic orientation towards metabolic abnormalities in young people [3,5,16]. This age criterion, to trigger a metabolic assessment in patients without sarcomeric mutations, could limit the costs linked to these biological assessments. Indeed, like any test, the search for an inherited metabolic disease must be motivated and integrated into a diagnostic process, especially since this research has a significant cost. This process must follow ESC recommendations, starting with eliminating the secondary causes of left ventricular hypertrophy (hypertension, hypertrophy caused by intense athletic training, etc.). Then, a genetic analysis of sarcomeric mutations should be carried out. Then, the tests should be directed towards other genetic and non-genetic etiologies according to clinical, electrocardiography, and echocardiography data. Finally, and only, a metabolic assessment can be considered after advice from a referral center. To summarize, metabolic research should not be carried out systematically and as a first intention. Thus, it is not possible to generalize tests to all patients with left ventricular hypertrophy.

Milder forms of IMD can be diagnosed during investigation of multisystem diseases, but in rare cases, the heart is the only affected organ and the main determinant of prognosis [1,17]. In some cases, specific therapy can slow the progression of the cardiac disease [18,19]. A permanent implantable cardiac device must be considered to prevent sudden death [20]. Fabry disease should be systematically screened because of the important risk of cardiac sudden death and it may benefit from specific treatment, which can stabilize renal function and decrease left ventricular mass when used at an early stage of the disease [21]. There are limited and discordant data about the prevalence of Fabry disease in patients with HCM (from 0% to 3%–4%). A recent FOCUS study reports a prevalence of 1.5% [22], similar to the results obtained by Monserrat et al. (1%) [11]. This data is in line with our study, which shows a 0.93% prevalence of Fabry disease in selected patients with HCM and a 1.04% in selected patients with cardiac rhythm abnormalities. Fabry disease can mimic sarcomeric HCM without extracardiac manifestations [23]. In accordance with this, we report the case of the patient diagnosed in the CardioMetabo study with a so-called cardiac variant of FD who presented an HCM and severe arrhythmia without extracardiac FD manifestations. Interestingly, this man’s heart symptomatology began with severe conduction disorder requiring PM implantation. Initially modest, HCM was later diagnosed. The delay of 12 years had to be noted between the onset of conduction disorders and Fabry disease diagnosis. This suggests that most patients with unexplained rhythm or conduction disorders should be screened for Fabry disease.

Fatty-acid metabolism disorders revealed by adult cardiac disease are rare but potentially lethal. Some cases, including medium-chain acyl-CoA dehydrogenase (MCAD) deficiency, were reported [6,7,24,25,26]. These cases show that a revelation of MCAD deficiency in adulthood (between 16 and 45 years of age) by a very severe acute clinical presentation associated with arrhythmia is possible. Adult (32 year-old) presentation of very long-chain acyl-CoA dehydrogenase (VLCAD) deficiency with cardiomyopathy, hypoglycemia, and rhabdomyolysis highlights a potential risk of cardiac complications even in adults [27]. The main problem is that fatty-acid metabolism disorders are the most difficult to identify because of the transient nature of clinical and biological manifestations [26]. It is therefore important to perform a metabolic assessment in acute disease [28]. We report here one case of SCAD deficiency identified in the CardioMetabo study. This young woman presented an HCM and a severe arrhythmia as well as renal failure and a medical history of seizures. The deficiency in SCAD is a poorly defined entity. Clinic expression is polymorph and nonspecific: the most frequent symptoms are chronic and episodic neuromuscular disabilities, hypotonia, and seizures [29,30]. The reported mutations are multiple and its physiopathology is still insufficiently understood [31]. It should be noted that individuals with SCAD deficiency could be asymptomatic [32]. As SCAD is needed at the end of the beta-oxidation spiral, there may be gluconeogenesis and ketogenic capacity from the preceding steps of fatty-acid oxidation which are probably sufficient to meet cellular energy demands under non-stressed conditions. As previously reported, SCAD deficiency seems more to be a disease of oxidative stress rather than energy deficiency, which could add to it being a misunderstood fatty-acid metabolism disorder [33]. Considering our patient, it is difficult to confirm the imputability of this deficit in her cardiac disease. Even if an energy deficit is not significant, the toxicity of C4-carnitine to the heart may be raised.

Our review of patients’ medical records presenting HCM and/or cardiac rhythm abnormalities permitted us to identify three mitochondrial pathologies. In all cases, cardiac involvement started in adulthood, was not isolated, and was part of a typical systemic picture of the disease. In the majority of cases of mitochondrial cytopathy, cardiac involvement falls within the multisystemic framework [8,33]. Hypertrophic remodeling is a dominant pattern of cardiomyopathy in all forms of mitochondrial disease and can mimic HCM [34,35,36]. A comprehensive assessment should therefore be carried out to examine the other organs in the case of heart disease and, conversely, to look for cardiac disease in case of mitochondrial disease diagnosis [37]. Mitochondrial cardiomyopathy must be considered in the absence of known mitochondrial disease because it might be the first, or even the sole, clinical manifestation [8,33,38,39]. We report here the case of a patient with Leber hereditary optic neuropathy presenting with a complete atrioventricular block requiring permanent PM implantation. The literature reports mainly Wolf-Parkinson-White syndrome related to Leber disease [1,8,40,41,42]. This is the first case of atrioventricular block reported in Leber disease.

It is interesting to note the higher plasma level of cardiac biomarkers (troponin and NT-pro-BNP) in patients with IMD. Similar data were reported in Fabry disease [43,44]. Elevated plasma levels of NT-pro-BNP and troponin are associated with higher risk of cardiovascular events, heart failure and death [3]. This confirms the severity of cardiac involvement and the importance of risk stratification in patients with IMD.

Strengths of our study include the use of the 2014 ESC guidelines on diagnosis and management of hypertrophic cardiomyopathy to perform the prevalence study in a large cohort of patients, and our results of prevalence are consistent with these recommendations [3]. Thanks to the thorough analysis of the medical records, the missing genetic tests were carried out. Seven patients with sarcomeric HCM were identified. The targeted metabolic assessment was performed only on selected patients with unexplained cardiac involvement and allowed us to identify two new patients with IMD. Limitations of the study are that etiology was not identified in 43.5% of patients with HCM and 40.6% of patients with cardiac rhythm abnormalities. These data are higher than those of the ESC (25–30% of unknown cases) [3], but above all, give us an opening to expand the complement of metabolic balance. The heterogeneity of the group of patients with severe arrhythmias and conduction disorders requiring cardiac implantable electronic devices can be considered as a limitation. However, integrated into a diagnostic process, we show, in this work, the interest of looking for IMD.

## 5. Conclusions

Hypertrophic cardiomyopathy and cardiac rhythm abnormalities are common cardiac manifestations of many inherited metabolic errors in adults. If cardiac disease has an unexplained etiology, possible metabolic diseases should be looked for, especially those which may benefit from a specific treatment. Therefore, we recommend that the diagnostic approach of patients with unexplained cardiac involvement (hypertrophic cardiomyopathy and/or cardiac arrhythmias and conduction disorders) systematically includes screening for Fabry disease, acylcarnitine profile analysis, and redox potential measurement.

## Figures and Tables

**Table 1:** Clinical characteristics in patients with IMD.

IMD	Age of Diagnosis, Sex	Clinical Presentation	Age of Onset of Heart Symptoms	Cardiac Presentation	Treatment	Evolution of Cardiac Damage
Hurler disease (MPS I)	3 years, W	Dysmorphic facial characteristic, Short neck, Dorsal kyphosis, Dysostosis multiplex, Umbilical hernia, Splenomegaly, Growth retardation	20 years	Septum HCM, Aortic and mitral insufficiency	Urbanyl, Depakin, Kardegic, Furosemid	More decompensations during 2 last years
MELAS syndrome	25 years, M	Behavior problems, Psychomotor developmental delay, Ataxia, Seizures, Hearing loss, Stroke-like, Diabetes	32 years	HCM, Sinoatrial bloc	Depakin, Keppra, Tercian, Platelet antiagregants, Tahor	Died a 32 (cardiogenic shock caused by supraventricular tachycardia)
Friedreich’s ataxia Patient 1	7 years, W	Cerebellar ataxia, Hearing loss, Voice defects, Swallowing disorders, dysarthria, Diabetes	18 years	HCM	Mnesis, Corgard, Triatec, Levocarnil, Vitamins	Stable
Friedreich’s ataxia Patient 2	19 years, W	Combined cerebellar and proprioceptive ataxia, Weight-loss, Scoliosis, Hips length inegality, Spasmophilia, Hyperprolactinemia	22 years	HCM, Repolarization abnormalities and positive troponin	Ubiten, Riluzol	Stable
Leber hereditary optic neuropathy	16 years, M	Bilateral visual loss	46 years	Left bundle-branch block, Complete atrioventricular block	PM	Stable
Fabry disease	64 years, M	Syncope	50 years	HCM (IVS = 27 mm) Complete atrioventricular blocNonsustained ventricular tachycardia	PM then ICD, Migalastat	Stable
SCAD deficiency	18 years, W	End-stage renal failure, Seizures, Syncope	20 years	HCM (IVS = 15 mm) Complete atrioventricular bloc	PM, Dialysis, treatment, Renal transplantation	Stable

HCM = hypertrophic cardiomyopathy; ICD = implantable cardioverter defibrillator; IMD = inherited metabolic disease; IVS = interventricular septum; MELAS = mitochondrial encephalopathy with lactic acidosis and stroke-like episodes; MPS = mucopolysaccharidosis; PM = pacemaker; SCAD = short chain acyl-CoA dehydrogenase.

**Table 2:** Results of laboratory tests.

	a : IMD	b : Sarcomeric	c : Unexplained	*p*
*n*	7	7	72	
Age, years, mean ± SD	39. 8 ± 15.6	50.4 ± 13.4	54.1 ± 12.9	0.026
Free carnitine, mmoL/L, mean ± SD	49 ± 9.9	44.9 ± 10.5	41.9 ± 9.9	NS
Total carnitine, mmoL/L, mean ± SD	61.5 ± 12	52.3 ± 14.5	49 ± 11.9	NS
Lactic acid, mmoL/L, mean ± SD	1.9 ± 1.2	1.3 ± 0.5	1.3 ± 0.7	NS
Pyruvic acid, mmoL/L, mean ± SD	0.09 ± 0.08	0.08 ± 0.03	0.07 ± 0.04	NS
Ratio Lactate/Pyruvate, mean ± SD	18 ± 1.6	15.3 ± 3.7	18.3 ± 5.4	NS
Hydroxybutyrate (HOB), mmoL/L, mean ± SD	0.163 ± 0.161	0.061 ± 0.038	0.068 ± 0.066	a|vs. c : 0.015 * a|vs. b : 0.03 *
Acetoacetate (AA), mmoL/L, mean ± SD	0.098 ± 0.077	0.053 ± 0.024	0.056 ± 0.040	NS
Ratio HOB/AA, mean ± SD	1.5 ± 0.4	1.1 ± 0.5	1.1 ± 0.5	NS
Median creatine kinase, U/L (IQR)	128 (67–203)	107 (84–134)	96 (67–152)	NS
Blood glucose, mmoL/L, mean ± SD	6.4 ± 2.6 (4.7–11.7)	5.8 ± 1.9 (4.4–10.1)	5.6 ± 1.4 (3.9–11)	NS
Median NT-pro-BNP, ng/L (IQR)	1009 (374–1683)	321 (197–1011)	173.5 (62.5–549.5)	a|vs. c : 0.043 *
Median troponin, µg/L (IQR)	0.22 (0.114–1.635)	0.015 (0.015–0.03)	0.015 (0.015–0.015)	a|vs. c : 0.043 *
Positive troponin, %	71.43	14.29	12.50	a|vs. c : 0.005 *
Median creatinine, µmol/L (IQR)	88.6 (41.4–104)	78.9 (61.1–100)	79.4 (69.45–88.5)	NS

IMD = inherited metabolic diseases; IQR = interquartile range; NS = non-significant; NT-pro-BNP = N-terminal pro-B-type natriuretic peptide; SD = standard deviation; *p* = prevalence; * = when omnibus *p*-value was <0.05, a two by two post-hoc test comparison was carried out.
